# Analysing Inequalities in Colorectal Cancer Screening Using an Individual Socioeconomic Status Index

**DOI:** 10.3390/cancers16233940

**Published:** 2024-11-25

**Authors:** Paula Romeo-Cervera, Javier Martín-Pozuelo, Mercedes Vanaclocha-Espí, Marina Pinto-Carbó, Susana Castán-Cameo, Dolores Salas, Ana Molina-Barceló

**Affiliations:** 1 Foundation for the Promotion of Health and Biomedical Research (FISABIO), 46020 Valencia, Spainana.molina@fisabio.es (A.M.-B.); 2 Joint Research Unit UMH-FISABIO (StatSalut), Miguel Hernández University, 03202 Elche, Spain; 3 General Directorate of Public Health, 46020 Valencia, Spain

**Keywords:** social inequalities, cancer screening, colorectal cancer, individual socioeconomic status index

## Abstract

**Background/Objectives:** An individual socioeconomic status index (ISESI) was used to analyse inequalities in participation and colonoscopy acceptance in the Valencia Region Colorectal Cancer Screening Programme (VR-CRCSP). **Methods:** This is a cross-sectional study of men and women aged 50–69 who had been invited to participate in the VR-CRCSP as of February 2020 (N = 1,066,763). The variables included in the ISESI were nationality, employment status, disability, healthcare coverage, risk of vulnerability, and family size. The ISESI was categorised into quartiles (Qs), with Q4 corresponding to the lowest socioeconomic status (SES). Odds ratios (ORs) and 95% confidence intervals (CIs) were estimated using mixed logistic regression models. **Results:** The results showed that Q2 (OR = 1.30, CI = 1.28–1.33) and Q3 (OR = 1.07, CI = 1.05–1.09) are more likely to participate in the CRCSP than Q1 (the highest SES), and that Q4 (OR = 0.77, CI = 0.76–0.78) is less likely to participate than Q1. In addition, Q2 (OR = 2.03, CI = 1.78–2.32), Q3 (OR = 1.90, CI = 1.67–2.16), and Q4 (OR = 1.55, CI = 1.36–1.76) are more likely to accept a colonoscopy than Q1. The following socioeconomic characteristics were related to both non-participation and colonoscopy refusal: not Spanish, disabled, no family unit, at risk of social vulnerability, and private mutual health insurance. **Conclusions:** Inequalities were observed in VR-CRCSP participation and colonoscopy acceptance.

## Simple Summary

This study uses an individual socioeconomic status index to analyse social inequalities in the Valencia Region Colorectal Cancer Screening Programme. Analysing the social determinants of cancer and ensuring health equity are some of the main challenges faced by developed countries in recent years, and these aspects are also the main focus of policies in many territories. Furthermore, given that the European Commission has highlighted the importance of evaluating inequalities in colorectal cancer (CRC) screening programmes, this study is highly relevant. This paper contributes to the existing literature on inequalities in cancer screening, and the insights provided herein could potentially influence policies and interventions to promote equity in CRC screening programmes.

## 1. Introduction

Colorectal cancer (CRC) is the second most common type of cancer and the second leading cause of cancer deaths in Europe, with approximately 520,000 new cases diagnosed and 245,000 deaths recorded in 2020 [1]. In Spain, CRC is the most common type of cancer, and an estimated 43,581 new cases were diagnosed in 2021 [2]. In terms of mortality, it is one of the main causes of cancer death in the population as a whole [2].

Early detection is one of the main strategies for reducing CRC incidence and mortality. As a result, in line with the recommendations of the Council of the European Union [3], population-based CRC early detection programmes have been implemented in most European countries. These programmes, which target the asymptomatic population with an average risk of developing colorectal cancer due to age and sex (men and women aged 50–69), use the faecal immunochemical test (FIT) as a screening test and offer a colonoscopy as a diagnostic test [4]. These programmes must be planned and delivered from a public health perspective and, consequently, should guarantee equal access [4,5].

The impact of CRC screening programmes (CRCSPs) on incidence and mortality can be measured in the long term. Nonetheless, standardised evaluation indicators and recommendations have also been developed to assess their effects in the short and medium term [4]. Some of these indicators are related to programme uptake and adherence. According to the European guidelines for quality assurance in CRC screening, these indicators should be evaluated for different socioeconomic characteristics to promote equity in screening programmes [4]. The European Commission advises a minimum uptake rate of 45% and a desirable rate of 65% and also recommends that the population have equitable access to these programmes [4].

The literature shows that CRC screening uptake is higher among women and older population groups [6], while there are mixed results in terms of socioeconomic status (SES). Some studies show a clear gradient in participation—i.e., the lower the SES, the lower the uptake [6]—while others find that uptake is lower in populations with the most extreme SES (both high and low) [7,8]. Differences have also been observed in colonoscopy acceptance. In Europe, it has been suggested that people with a lower SES are less likely to accept a colonoscopy [6,9,10,11]. On the contrary, some studies carried out in Spain have observed the opposite and reveal that people with a higher SES are less likely to accept a colonoscopy [6,8,12].

Previous studies have attributed inequalities in CRCSPs to the presence of economic, social, and cultural barriers in the population [8,13]. These barriers may prevent socially vulnerable population groups from accessing early detection initiatives, thus increasing their risk of developing advanced CRC and generating social inequalities in health [14,15].

Health inequalities are defined as unjust, avoidable, and unnecessary systematic differences in the health status of different population groups [16]. In turn, social disparities in cancer are inequalities that span the cancer continuum throughout the life cycle [17]. In accordance with the recommendations of the European Commission, cancer screening programmes must guarantee equity, and any inequalities should be evaluated [4,5]. At present, few CRCSPs evaluate the impact of social inequalities, and those that do so normally use a territorial socioeconomic status index [7,8,18].

The Valencia Region (Spain) Colorectal Cancer Screening Programme (VR-CRCSP) is a population-based programme aimed at men and women aged 50–69. Every two years, it offers a faecal immunochemical test (FIT) and a colonoscopy as a confirmatory diagnostic test [19]. The programme is in line with the European Guidelines [4]. Uptake is around 45% [19], and inequalities in participation are not analysed on a regular basis.

The Population Information System of the Valencia Region (PIS) contains up-to-date socioeconomic data on all individuals residing in the Valencia Region, including participants in the VR-CRCSP. For this study, these data were aggregated into an individual socioeconomic status index (ISESI) with the aim of measuring socioeconomic inequalities in CRCSP indicators [12].

Therefore, taking into account European recommendations to ensure equity and evaluate any inequalities in CRCSP, this study aims to use an ISESI to analyse social inequalities in participation and colonoscopy acceptance in the VR-CRCSP.

## 2. Materials and Methods

### 2.1. Study Design and Population

This is a cross-sectional, observational study from a retrospective cohort of men and women aged 50–69 who had been invited to participate in the last round of the Valencia Region (Spain) Colorectal Cancer Screening Programme (VR-CRCSP) as of February 2020. A programme round is the 2-year period during which the target population is invited to participate.

Any individuals with missing data on the socioeconomic variables used to create the index were excluded from the study. The study population totalled 1,066,763 people.

### 2.2. Data Sources and Study Variables

The data analysed in this study were obtained from the VR-CRCSP information system, which contains personal data and information regarding screening invitations and participation outcomes. In turn, this information system periodically draws on the socioeconomic data contained in the PIS.

Socioeconomic information from the PIS was used to develop an ISESI for the population aged between 50 and 75 based on six individual socioeconomic characteristics aggregated into a single quantitative index, following the methodology described by Vanaclocha-Espí et al. [12]. Low ISESI values are related to a more disadvantaged socioeconomic status.

The variables included in the ISESI are nationality (Spanish/not Spanish), employment status (retired/employed/unemployed), disability (disabled: with a degree of disability of ≥65%/not disabled), healthcare coverage (social security/public mutual health insurance/private mutual health insurance/European Health Insurance Card healthcare coverage), risk of vulnerability (no risk/risk due to unemployment: unemployed people/risk due to low income: people without any income, including foreign immigrants without legal residence), and family size (small: <3 people/medium: 3–4 people/large: >4 people/no family unit: institutionalised people) [12].

The outcome variables are global participation (yes/no; the “yes” category is for individuals invited to take part in the CRCSP who actually performed and delivered the FIT), initial participation (yes/no; this variable is measured for people who have been invited but have not previously participated in the programme), subsequent participation (yes/no; this variable is measured for people who have been invited and have previously participated in the programme), and colonoscopy acceptance (yes/no; the “yes” category refers to individuals who underwent a diagnostic colonoscopy after receiving a positive FIT result).

The main explanatory variable was the ISESI calculated for each individual [12]. This numerical index was categorised into four groups based on quartiles (Qs), where Q1 corresponds to the most privileged socioeconomic characteristics and Q4 to the most disadvantaged socioeconomic characteristics.

Other explanatory variables were sex (men/women), age (50–54/55–59/60–64/65–69 years old), type of invitation (initial screening, first invitation: people being invited to participate in the CRCSP for the first time/subsequent invitation for previous never-responders: people who had been invited to previous rounds but did not participate/subsequent invitation, regular intervals: people who had been invited to the previous round and participated/subsequent invitation, irregular intervals: people who had been invited to the immediately previous round and did not participate, but who had participated in at least one round previously), and health department.

### 2.3. Statistical Analysis

The frequencies and percentages for outcome variables according to the ISESI and ISESI variables were used to carry out a descriptive study. The chi-square test was used to analyse the relationship between outcome and explanatory variables. In order to assess the effect of the ISESI on outcome variables, mixed logistic regression models were fitted considering age, sex, type of invitation (when analysing global participation), and health department, with the last variable as a random effect. The models were fitted for the overall sample and were stratified by sex and age group (50–59 and 60–69 years old).

The same methodology was used to study the relationship between each of the six ISESI variables and the outcome variables. In this case, independent models were applied for each ISESI variable. The results of the models are shown in terms of odds ratios (ORs) and 95% confidence intervals (CIs). A significance level of 0.05 was established and used to interpret the results. All analyses were performed with the statistical software programme R v4.1.1.

### 2.4. Ethical Considerations

This study was approved by the Research Ethics Committee of the General Directorate of Public Health and the Advanced Public Health Research Centre (CEI GDSP-CSISP) in Decision No. 20240126/08/E. The personal data included in this study were pseudonymised to guarantee the confidentiality, privacy, and security thereof. All project research staff declared that they have no conflicts of interest with the project. The project was carried out in accordance with the principles of the Declaration of Helsinki and the requirements of Spanish data confidentiality legislation (Organic Law 3/2018 of 5 December on personal data protection and the guarantee of digital rights). The ethics committee issued an exemption from the requirement to obtain informed consent as this is an observational study with a large sample (1,000,000 people), in accordance with the provisions of the current Declaration of Helsinki.

## 3. Results

The descriptive results (Table 1) show that the lowest CRCSP participation rate is found in Q4 (global participation = 38.67%, initial participation = 19.07%, subsequent participation = 87.41%) and the highest participation rate is found in Q2 (global participation = 53.96%, initial participation = 29.62%, subsequent participation = 90.96%), with statistically significant differences. In addition, the results show higher colonoscopy acceptance in Q2 (90.59%), Q3 (89.00%), and Q4 (88.47%) than in Q1 (83.86%), also with statistically significant differences. All ISESI variables show statistically significant differences in terms of participation and colonoscopy acceptance.

The global participation models (Table 2) show that Q2 (OR = 1.30, CI = 1.28–1.33) and Q3 (OR = 1.07, CI = 1.05–1.09) are more likely to participate than the reference group (Q1: best SES) and that Q4 is less likely to participate than the reference group. Similar results can be observed in the initial participation models (Q2: OR = 1.34, CI = 1.31–1.36/Q3: OR = 1.04, CI = 1.02–1.06/Q4: OR = 0.76, CI = 0.75–0.77) and subsequent participation models (Q2: OR = 1.19, CI = 1.15–1.23/Q3: OR = 1.12, CI = 1.08–1.16/Q4: OR = 0.85, CI = 0.82–0.88). The results are similar when stratified by sex and age, with a stronger association in men (Q2: OR = 1.52, CI = 1.48–1.56/Q4: OR = 0.65, CI = 0.63–0.66) and the 50–59 age group (Q2: OR = 1.40, CI = 1.37–1.42/Q3: OR = 0.93, CI = 0.91–0.95/Q4: OR = 0.75, CI = 0.74–0.76).

The colonoscopy acceptance models (Table 2) show that Q2 (OR = 2.03, CI = 1.78–2.32), Q3 (OR = 1.90, CI = 1.67–2.16), and Q4 (OR = 1.55, CI = 1.36–1.76) are more likely to accept a colonoscopy than Q1. The models provide similar results when stratified by sex and age, with a stronger association in men (Q2: OR = 2.24, CI = 1.84–2.73/Q3: OR = 2.06, CI = 1.74–2.44) and the 60–69 age group (Q2: OR = 2.97, CI = 2.42–3.65/Q3: OR = 3.19, CI = 2.67–3.80/Q4: OR = 2.65, CI = 2.16–3.26).

The models studying the relationship between participation and the different socioeconomic variables included in the ISESI (Table 3) show lower participation in non-Spanish population groups (OR = 0.53, CI = 0.52–0.54), unemployed people (OR = 0.67, CI = 0.66–0.68), retired people (OR = 0.85, CI = 0.84–0.87), disabled people (OR = 0.63, CI = 0.62–0.65), people with private mutual health insurance (OR = 0.82, CI = 0.79–0.84) or European Health Insurance Card healthcare coverage (OR = 0.33, CI = 0.26–0.42), and the population at risk of social vulnerability due to low income (OR = 0.51, CI = 0.49–0.52) or unemployment (OR = 0.78, CI = 0.76–0.79), in addition to those with a large family unit (OR = 0.92, CI = 0.90–0.94) or no family unit (OR = 0.48, CI = 0.46–0.50). Similar results are observed when the sample is stratified by sex and age. The same results can be seen for initial and subsequent participation (Tables S1 and S2), with the only difference being that retirement increases the probability of subsequent participation in men (OR = 1.09, CI = 1.03–1.15) and the 60–69 age group (OR = 1.24, CI = 1.17–1.31).

The models studying the relationship between colonoscopy acceptance and the ISESI variables (Table 4) show a higher probability of refusal in the non-Spanish population (OR = 0.80, CI = 0.67–0.95), disabled people (OR = 0.46, CI = 0.39–0.43), people with private mutual health insurance (OR = 0.15, CI = 0.13–0.18) or European Health Insurance Card healthcare coverage (OR = 0.15, CI = 0.05–0.46), the population at risk due to low income (OR = 0.73, CI = 0.59–0.90), and people with no family unit (OR = 0.39, CI = 0.29–0.50). No relationship was found between employment status and colonoscopy acceptance. Similar results were observed when the sample was stratified by sex and age.

## 4. Discussion

The results of this study show that the VR-CRCSP is affected by social inequalities in terms of participation and colonoscopy acceptance and that SES is a significant determinant in these inequalities.

With regard to participation, the results show that both initial and subsequent participation is higher in the medium quartiles than in the extreme SES groups (best and worst SES). Most studies conducted in the context of European CRCSP reveal a gradient in participation, with lower participation among people from less privileged social strata [6,20,21,22], while other studies carried out in the Spanish context are in line with our findings and show that uptake is lower at the extremes of the social scale, i.e., both lower and higher class groups have lower participation rates [7,8].

This study also found that the ISESI has a greater impact on initial participation than subsequent participation. This is because it is more difficult for an individual to leave the screening circuit once they have already participated in the programme, and therefore, the ISESI does not have such a significant impact [23]. Accordingly, social inequalities must be addressed as soon as the population becomes eligible to participate as it is easier to reduce these inequalities once participants are involved in the CRCSP.

Regarding sex, this study shows that the ISESI has a greater impact on the participation of men and acts as a facilitator for individuals in Q2 and a barrier for those in Q4. This variable impact can be partly attributed to traditional gender roles, as discussed in Ana Molina et al., which suggests that economic stability and educational level are crucial factors in health-promoting behaviours in men [13]. With regard to differences between men and women, other studies suggest that men, particularly those from lower socioeconomic backgrounds, are less likely to participate than women [10,13].

This study also analyses the profile of the population with lower participation rates. This population is characterised by the most disadvantaged socioeconomic characteristics, such as being unemployed, disabled, at risk of vulnerability due to low income, and living in a large family unit or having no family unit, and the most privileged socioeconomic characteristics, such as having private mutual health insurance or European Health Insurance Card healthcare coverage. This is in line with the findings of the ISESI, where extreme quartiles have lower participation.

Regarding the lower participation of the disabled population, these results are in line with the study conducted by Kellen E. et al. [24] in Belgium, which showed that the CRC screening participation rate of disabled individuals was 10% lower than the Flemish average during the same period.

It has been observed that the population at risk due to low income (people without any income, including foreign immigrants without legal residence) also has a lower CRCSP participation rate. Immigrants often have lower screening rates compared to native-born individuals, and this difference could be due to the fact that immigrant populations face several barriers to gaining access to screening programmes, including language difficulties, cultural differences, a lower SES, and a lack of tailored health promotion efforts [25].

This study reveals that unemployed individuals also have a lower CRCSP participation rate. Similar results were observed in other studies conducted in Europe that found lower CRCSP participation rates among unemployed people than among employed people [26,27].

Regarding the impact of SES on CRCSP participation, the lower uptake observed in the population with a higher SES in this study may be due to the fact that more affluent individuals use private healthcare services for screening tests, as has been observed in other screening programmes [28,29]. This coincides with the study carried out by Buron et al. [8], which found that uptake was higher in the middle quintiles than in the least deprived and most deprived quintiles. The same is true for colonoscopy acceptance, where all quartiles show a higher acceptance rate than Q1 regardless of age and sex. As with participation, this may be due to the fact that more socially privileged groups leave the public healthcare circuit when they receive a positive FIT result and undergo a colonoscopy in a private healthcare setting, and not because they reject the colonoscopy per se. This can be explained by the fact that the Spanish public healthcare system is universal and free for the entire population but private health insurance is also available, although this is usually only an option for people with a higher SES. Some studies have opposing results and find lower colonoscopy acceptance following a positive FIT result among people with a lower SES [10,11,14,30].

In this regard, the results of this study show that the type of healthcare coverage influences participation and colonoscopy acceptance. Having private health insurance reduces the likelihood of participating in the CRCSP and accepting a colonoscopy. Furthermore, as mentioned before, people with private mutual health insurance may have lower participation and acceptance rates due to the fact that they leave the public healthcare circuit in favour of private healthcare services.

One of the strengths of this study is that it uses an individual SES index to analyse inequalities in CRC screening. The majority of existing studies analysed the relationship between SES and participation from an ecological point of view using territorial SES indices [7,8,18], whereas we used an individual index. We cannot analyse inequalities in cancer without socioeconomic indicators. However, such indicators are not always readily available at the population level. For this reason, most studies analyse inequalities in CRC screening using territorial indicators of social deprivation [7,8,18,31]. In contrast to this type of index, which can camouflage inequalities in socioeconomically diverse areas, the ISESI used in this study allows us to analyse inequalities associated with individuals.

Additionally, this study reveals that SES has a greater impact on men and younger age groups, a new finding that contributes to existing evidence on social inequalities in cancer prevention.

Using an ISESI to systematically analyse inequalities in screening programmes is a good practice in accordance with European Commission recommendations, which prioritise monitoring and evaluating the quality and equity of screening programmes [4,5].

## 5. Conclusions

Inequalities are observed in CRC screening uptake and colonoscopy acceptance. To promote social equity in early detection, we must first identify any social inequalities in CRC screening. The results of this study are a starting point for promoting CRC screening interventions, policies, and programmes based on the principle of proportional universalism, focusing on the needs of different social groups to reduce inequalities in CRC incidence and mortality.

## Figures and Tables

**Table 1:** Participation and colonoscopy acceptance for the ISESI quartiles and different socioeconomic characteristics (percentage % and nominator).

	Global Participation	Initial Participation	Subsequent Participation	Colonoscopy Acceptance
	% (*n*)	% (*n*)	% (*n*)	% (*n*)
	47.51 (506,770)	23.05 (155,798)	89.80 (350,972)	88.76 (22,821)
ISESI				
Q1 (best SES)	45.43 (70,000)	25.06 (26,349)	89.21 (43,651)	83.86 (2400)
Q2	53.96 (161,614)	29.62 (53,508)	90.96 (108,106)	90.59 (6555)
Q3	49.81 (170,009)	19.86 (38,960)	90.32 (131,049)	89.00 (8969)
Q4 (worst SES)	38.67 (105,147)	19.07 (36,981)	87.41 (68,166)	88.47 (4897)
*p*-value	<0.001	<0.001	<0.001	<0.001
Sex				
Men	45.59 (232,840)	22.12 (73,640)	89.59 (159,200)	89.08 (12,741)
Women	49.26 (273,930)	23.96 (82,158)	89.97 (191,772)	88.37 (10,080)
*p*-value	<0.001	<0.001	<0.001	0.070
Age (years)				
50–59	44.71 (254,430)	26.90 (109,441)	89.30 (145,019)	90.08 (10,286)
60–69	50.70 (252,340)	17.23 (46,387)	90.15 (205,953)	87.71 (12,535)
*p*-value	<0.001	<0.001	<0.001	<0.001
Nationality				
Spanish	48.85 (484,119)	23.66 (145,920)	90.31 (338,199)	88.88 (21,756)
Not Spanish	29.94 (22,651)	16.66 (9878)	78.08 (12,773)	86.44 (1065)
*p*-value	<0.001	<0.001	<0.001	<0.001
Employment status				
Employed	48.72 (180,096)	30.22 (76,913)	89.62 (103,183)	90.18 (6834)
Unemployed	39.31 (75,209)	20.62 (28,601)	88.54 (46,608)	89.69 (3133)
Retired	49.72 (251,465)	17.79 (50,284)	90.18 (201,181)	87.81 (12,854)
*p*-value	<0.001	<0.001	<0.001	<0.001
Disability				
Not disabled	47.95 (494,103)	23.35 (151,753)	89.98 (342,350)	89.14 (22,048)
Disabled	34.82 (12,667)	15.56 (4045)	83.03 (8622)	79.12 (773)
*p*-value	<0.001	<0.001	<0.001	<0.001
Healthcare coverage				
Social security	47.69 (485,317)	23.17 (148,993)	89.80 (336,324)	89.60 (22,298)
Public mutual health insurance	58.92 (5497)	33.36 (1754)	91.92 (3743)	87.19 (177)
European Health Insurance Card	21.63 (141)	10.94 (58)	68.03 (83)	53.85 (7)
Private mutual health insurance	40.44 (15,815)	18.50 (4993)	89.27 (10,822)	55.76 (339)
*p*-value	<0.001	<0.001	<0.001	<0.001
Risk of vulnerability				
No risk	48.97 (451,249)	23.81 (136,083)	90.06 (315,166)	88.70 (20,267)
Risk due to low income	28.63 (10,571)	13.71 (3958)	82.04 (6613)	85.08 (593)
Risk due to unemployment	41.48 (44,950)	20.87 (15,757)	88.83 (29,193)	90.66 (1961)
*p*-value	<0.001	<0.001	<0.001	<0.001
Family size				
Small	45.92 (217,594)	20.06 (59,581)	89.34 (158,013)	87.90 (10,050)
Medium	51.48 (238,518)	27.42 (78,981)	91.05 (159,537)	89.81 (10,487)
Large	41.70 (45,396)	20.45 (15,201)	87.45 (30,195)	89.57 (2060)
No family unit	25.40 (5262)	12.35 (2035)	76.00 (3227)	74.67 (224)
*p*-value	<0.001	<0.001	<0.001	<0.001

ISESI, individual socioeconomic status index; Q, quartile; SES, socioeconomic status.

**Table 2:** Results of the ISESI models for the overall sample and stratified by sex and age.

Process Indicators		Overall Sample	Men	Women	50–59 Years Old	60–69 Years Old
		OR (CI 95%)				
Participation	ISESI: Q1 (best SES)	Ref.	Ref.	Ref.	Ref.	Ref.
	Q2	1.30 (1.28–1.33)	1.52 (1.48–1.56)	1.23 (1.20–1.26)	1.40 (1.37–1.42)	1.18 (1.13–1.22)
	Q3	1.07 (1.05–1.09)	1.04 (1.01–1.07)	0.98 (0.96–1.01)	0.93 (0.91–0.95)	1.11 (1.08–1.15)
	Q4 (worst SES)	0.77 (0.76–0.78)	0.65 (0.63–0.66)	0.87 (0.85–0.89)	0.75 (0.74–0.76)	0.81 (0.78–0.84)
Initial participation	ISESI: Q1 (best SES)	Ref.	Ref.	Ref.	Ref.	Ref.
	Q2	1.34 (1.31–1.36)	1.58 (1.54–1.62)	1.24 (1.21–1.27)	1.48 (1.46–1.51)	1.11 (1.07–1.16)
	Q3	1.04 (1.02–1.06)	0.97 (0.95–1.00)	1.00 (0.97–1.03)	0.90 (0.88–0.92)	1.06 (1.02–1.11)
	Q4 (worst SES)	0.76 (0.75–0.77)	0.65 (0.63–0.67)	0.84 (0.82–0.86)	0.71 (0.70–0.72)	0.87 (0.83–0.91)
Subsequent participation	ISESI: Q1 (best SES)	Ref.	Ref.	Ref.	Ref.	Ref.
	Q2	1.19 (1.15–1.23)	1.26 (1.19–1.33)	1.19 (1.14–1.25)	1.25 (1.20–1.31)	1.18 (1.11–1.26)
	Q3	1.12 (1.08–1.16)	1.16 (1.10–1.22)	0.99 (0.94–1.04)	0.90 (0.86–0.95)	1.14 (1.08–1.21)
	Q4 (worst SES)	0.85 (0.82–0.88)	0.68 (0.64–0.72)	0.99 (0.94–1.04)	0.90 (0.86–0.94)	0.75 (0.71–0.80)
Colonoscopy acceptance	ISESI: Q1 (best SES)	Ref.	Ref.	Ref.	Ref.	Ref.
	Q2	2.03 (1.78–2.32)	2.24 (1.84–2.73)	1.78 (1.47–2.17)	1.59 (1.34–1.90)	2.97 (2.42–3.65)
	Q3	1.90 (1.67–2.16)	2.06 (1.74–2.44)	1.62 (1.32–1.99)	1.03 (0.84–1.25)	3.19 (2.67–3.80)
	Q4 (worst SES)	1.55 (1.36–1.76)	1.47 (1.24–1.75)	1.66 (1.36–2.03)	1.13 (0.95–1.34)	2.65 (2.16–3.26)

ISESI, individual socioeconomic status index; SES, socioeconomic status; Q, quartile. Overall sample models adjusted for age, sex, type of invitation, and health department.

**Table 3:** Results of the models relating participation with each ISESI variable for the overall sample and stratified by sex and age.

Participation		Overall Sample ^1^	Men ^2^	Women ^2^	50–59 Years Old ^3^	60–69 Years Old ^3^
		OR (CI 95%)	OR (CI 95%)	OR (CI 95%)	OR (CI 95%)	OR (CI 95%)
Nationality	Spanish	Ref.	Ref.	Ref.	Ref.	Ref.
	Not Spanish	0.53 (0.52–0.54)	0.49 (0.47–0.50)	0.56 (0.55–0.58)	0.44 (0.43–0.45)	0.68 (0.66–0.71)
Employment status	Employed	Ref	Ref.	Ref.	Ref.	Ref.
	Unemployed	0.67 (0.66–0.68)	0.51 (0.49–0.52)	0.78 (0.76–0.79)	0.65 (0.64–0.66)	0.82 (0.78–0.86)
	Retired	0.85 (0.84–0.87)	0.85 (0.83–0.87)	0.86 (0.84–0.88)	0.76 (0.75–0.78)	1.01 (0.98–1.05)
Disability	Not disabled	Ref.	Ref.	Ref.	Ref.	Ref.
	Disabled	0.63 (0.62–0.65)	0.67 (0.64–0.69)	0.61 (0.58–0.63)	0.64 (0.61–0.67)	0.63 (0.60–0.66)
Healthcare coverage	Social security	Ref.	Ref.	Ref.	Ref.	Ref.
	Public mutual health insurance	1.49 (1.41–1.58)	1.52 (1.40–1.65)	1.46 (1.35–1.57)	1.55 (1.44–1.66)	1.38 (1.27–1.51)
	European Health Insurance Card	0.33 (0.26–0.42)	0.41 (0.29–0.59)	0.29 (0.21–0.39)	0.57 (0.14–2.34)	0.31 (0.25–0.40)
	Private mutual health insurance	0.82 (0.79–0.84)	0.86 (0.82–0.90)	0.78 (0.75–0.81)	0.79 (0.76–0.82)	0.85 (0.81–0.88)
Risk of social vulnerability	No risk	Ref.	Ref.	Ref.	Ref.	Ref.
	Risk due to low income	0.51 (0.49–0.52)	0.44 (0.42–0.47)	0.56 (0.53–0.58)	0.50 (0.48–0.52)	0.53 (0.51–0.56)
	Risk due to unemployment	0.78 (0.76–0.79)	0.64 (0.62–0.66)	0.90 (0.88–0.92)	0.78 (0.76–0.79)	0.80 (0.76–0.83)
Family size	Small	Ref.	Ref.	Ref.	Ref.	Ref.
	Medium	1.31 (1.29–1.32)	1.38 (1.36–1.40)	1.24 (1.22–1.26)	1.48 (1.46–1.50)	1.09 (1.07–1.11)
	Large	0.92 (0.90–0.94)	0.95 (0.92–0.97)	0.89 (0.87–0.92)	0.99 (0.97–1.01)	0.86 (0.83–0.88)
	No family unit	0.48 (0.46–0.50)	0.50 (0.48–0.53)	0.60 (0.44–0.49)	0.50 (0.47–0.53)	0.48 (0.45–0.51)

^1^ Overall sample models adjusted for age, sex, type of invitation, and health department. ^2^ Models adjusted for age, type of invitation, and health department. ^3^ Models adjusted for sex, type of invitation, and health department

**Table 4:** Results of the colonoscopy acceptance models for each ISESI variable, for the overall sample and stratified by sex and age.

Colonoscopy Acceptance		Overall Sample ^1^	Men ^2^	Women ^2^	50–59 Years Old ^3^	60–69 Years Old ^3^
		OR (CI 95%)	OR (CI 95%)	OR (CI 95%)	OR (CI 95%)	OR (CI 95%)
Nationality	Spanish	Ref.	Ref.	Ref.	Ref.	Ref.
	Not Spanish	0.80 (0.67–0.95)	0.75 (0.59–0.96)	0.86 (0.66–1.12)	0.63 (0.48–0.82)	0.92 (0.73–1.17)
Employment status	Employed	Ref.	Ref.	Ref.	Ref.	Ref.
	Unemployed	0.97 (0.85–1.11)	0.87 (0.71–1.07)	1.08 (0.90–1.31)	0.91 (0.78–1.05)	1.64 (1.14–2.36)
	Retired	0.94 (0.81–1.08)	0.95 (0.78–1.14)	0.94 (0.76–1.17)	0.77 (0.65–0.92)	1.23 (0.99–1.52)
Disability	Not disabled	Ref.	Ref.	Ref.	Ref.	Ref.
	Disabled	0.46 (0.39–0.54)	0.42 (0.34–0.53)	0.51 (0.40–0.64)	0.46 (0.35–0.60)	0.46 (0.37–0.56)
Healthcare coverage	Social security	Ref.	Ref.	Ref.	Ref.	Ref.
	Public mutual health insurance	0.80 (0.53–1.22)	0.78 (0.45–1.36)	0.81 (0.43–1.54)	0.75 (0.40–1.41)	0.84 (0.48–1.45)
	European Health Insurance Card	0.15 (0.05–0.46)	0.18 (0.04–0.81)	0.15 (0.03–0.74)		0.18 (0.06–0.57)
	Private mutual health insurance	0.15 (0.13–0.18)	0.15 (0.12–0.19)	0.15 (0.11–0.19)	0.14 (0.11–0.19) *	0.16 (0.13–0.19)
Risk of social vulnerability	No risk	Ref.	Ref.	Ref.	Ref.	Ref.
	Risk due to low income	0.73 (0.59–0.90)	0.56 (0.40–0.79)	0.86 (0.65–1.13)	0.60 (0.44–0.81)	0.85 (0.63–1.16)
	Risk due to unemployment	1.12 (0.96–1.31)	1.06 (0.84–1.32)	1.19 (0.96–1.48)	1.12 (0.93–1.34)	1.18 (0.89–1.58)
Family size	Small	Ref.	Ref.	Ref.	Ref.	Ref.
	Medium	1.14 (1.05–1.24)	1.16 (1.03–1.30)	1.12 (0.99–1.27)	1.23 (1.07–1.40)	1.10 (0.99–1.23)
	Large	1.12 (0.97–1.30)	1.20 (0.98–1.47)	1.07 (0.86–1.32)	1.13 (0.91–1.40)	1.18 (0.96–1.43)
	No family unit	0.39 (0.29–0.50)	0.39 (0.27–0.55)	0.39 (0.26–0.59)	0.42 (0.28–0.65)	0.37 (0.26–0.52)

* No cases in the European Health Insurance Card group. ^1^ Overall sample models adjusted for age, sex, type of invitation, and health department. ^2^ Models adjusted for age, type of invitation, and health department. ^3^ Models adjusted for sex, type of invitation, and health department

## Data Availability

The datasets presented in this article are not readily available due to legal restrictions on sharing the dataset, as regulated by the Valencia regional government by means of legal resolution by the Valencia Health Agency (2009/13312), which forbids the dissemination of data to third parties. Available from https://www.san.gva.es/ca/web/investigacio/programas-normativa-y-legislacion (accessed on 13 February 2024). Upon request, authors can obtain access to the databases to verify the accuracy of the analysis or the reproducibility of the study. Requests to access the datasets should be directed to the Management Office of the Data Commission of the Valencia Health Agency. Available from https://www.san.gva.es/ca/web/investigacio/acceso-a-la-aplicación (accessed on 13 February 2024).

## Supplementary Materials

The following supporting information can be downloaded at https://www.mdpi.com/article/10.3390/cancers16233940/s1, Table S1: Results of the models for initial participation with each ISESI variables, for the overall sample and stratified by sex and age; Table S2: Results of the models for subsequent participation with each ISESI variables, for the overall sample and stratified by sex and age.
