# Supplementary material for: Analysing Inequalities in Colorectal Cancer Screening Using an Individual Socioeconomic Status Index
Source: Cancers (Basel). 2024 Nov 25;16(23):3940. doi: 10.3390/cancers16233940 (PMC11640264; doi:10.3390/cancers16233940)
Supplement: Supplementary file 1 [file cancers-16-03940-s001.zip › cancers-3252138-supplementary.pdf]

Table S1. Results of the models for initial participation with each ISESI variables, for the overall sample and stratified by sex and age.

Initial participation		Overall sample[1]	Men[2]	Women[2]	50-59 years[3]	60-69 years[3]
		OR (CI 95%)	OR (CI 95%)	OR (CI 95%)	OR (CI 95%)	OR (CI 95%)
**Nacionality**	Spanish	Ref.	Ref.	Ref.	Ref.	Ref.
	Not spanish	0.65 (0.64-0.67)	0.59 (0.57-0.61)	0.71 (0.69-0.73)	0.50 (0.49-0.52)	1.06 (1.02-1.10)
**Employment status**	Employed	Ref.	Ref.	Ref.	Ref.	Ref.
	Unemployed	0.62 (0.61-0.63)	0.47 (0.46-0.49)	0.73 (0.71-0.74)	0.58 (0.57-0.69)	0.76 (0.72-0.81)
	Retired	0.77 (0.76-0.79)	0.77 (0.74-0.79)	0.78 (0.76-0.81)	0.63 (0.61-0.64)	0.81 (0.78-0.84)
**Disability**	Not disabled	Ref.	Ref.	Ref.	Ref.	Ref.
	Disabled	0.65 (0.63-0.67)	0.70 (0.66-0.73)	0.60 (0.58-0.64)	0.61 (0.59-0.64)	0.67 (0.63-0.71)
**Healthcare coverage**	Social security	Ref.	Ref.	Ref.	Ref.	Ref.
	Public mutualism	1.58 (1.49-1.68)	1.63 (1.49-1.78)	1.54 (1.42-1.66)	1.57 (1.44-1.69)	1.53 (1.38-1.69)
	European Health	0.67 (0.51-0.88)	0.78 (0.52-1.18)	0.60 (0.41-0.87)	0.60 (0.13-2.65)	0.57 (0.43-0.75)
	Private mutualism	0.77 (0.74-0.79)	0.83 (0.79-0.87)	0.72 (0.69-0.75)	0.74 (0.71-0.77)	0.78 (0.74-0.82)
**Risk of vulnerability**	No risk	Ref.	Ref.	Ref.	Ref.	Ref.
	Risk due to low	0.51 (0.49-0.53)	0.45 (0.43-0.48)	0.55 (0.53-0.57)	0.49 (0.47-0.51)	0.55 (0.52-0.59)
	Risk due to	0.74 (0.73-0.76)	0.60 (0.59-0.62)	0.86 (0.84-0.88)	0.71 (0.69-0.72)	0.86 (0.81-0.90)
**Family size**	Small	Ref.	Ref.	Ref.	Ref.	Ref.
	Medium	1.33 (1.31-1.34)	1.40 (1.38-1.43)	1.26 (1.23-1.28)	1.57 (1.55-1.60)	1.05 (1.03-1.07)
	Large	0.94 (0.92-0.96)	0.96 (0.94-0.99)	0.91 (0.89-0.94)	1.06 (1.03-1.08)	0.85 (0.82-0.88)
	No family unit	0.53 (0.51-0.56)	0.57 (0.54-0.61)	0.50 (0.46-0.53)	0.54 (0.50-0.57)	0.56 (0.52-0.61)

ISESI, individual socioeconomic status index; SES, socioeconomic status; Q, quartile.
Overall sample models adjusted for age, sex, type of invitation and health department.

Table S2. Results of the models for subsequent participation with each ISESI variables, for the overall sample and stratified by sex and age.

Subsequent participation		Overall sample[1]	Men[2]	Women[2]	50-59 years[3]	60-69 years[3]
		OR (CI 95%)	OR (CI 95%)	OR (CI 95%)	OR (CI 95%)	OR (CI 95%)
**Nacionality**	Spanish	Ref.	Ref.	Ref.	Ref.	Ref.
	Not spanish	0.42 (0.40-0.43)	0.39 (0.37-0.42)	0.43 (0.41-0.46)	0.38 (0.36-0.40)	0.44 (0.41-0.46)
**Employment status**	Employed	Ref.	Ref.	Ref.	Ref.	Ref.
	Unemployed	0.89 (0.86-0.92)	0.65 (0.62-0.69)	1.01 (0.97-1.06)	0.86 (0.83-0.90)	0.95 (0.88-1.03)
	Retired	1.04 (1.00-1.08)	1.09 (1.03-1.15)	1.00 (0.95-1.06)	0.85 (0.81-0.90)	1.22 (1.16-1.29)
**Disability**	Not disabled	Ref.	Ref.	Ref.	Ref.	Ref.
	Disabled	0.53 (0.51-0.56)	0.54 (0.50-0.58)	0.53 (0.49-0.53)	0.59 (0.54-0.64)	0.51 (0.48-0.55)
**Healthcare coverage**	Social security	Ref.	Ref.	Ref.	Ref.	Ref.
	Public mutualism	1.24 (1.10-1.38)	1.25 (1.06-1.48)	1.21 (1.04-1.41)	1.32 (1.11-1.56)	1.16 (1.00-1.35)
	European Health	0.32 (0.21-0.46)	0.39 (0.20-0.76)	0.29 (0.18-0.46)		0.31 (0.21-0.46)
	Private	0.95 (0.90-1.01)	0.96 (0.88-1.05)	0.94 (0.87-1.02)	0.94 (0.86-1.03)*	0.96 (0.89-1.03)
**Risk of vulnerability**	No risk	Ref.	Ref.	Ref.	Ref.	Ref.
	Risk due to low	0.50 (0.47-0.53)	0.43 (0.39-0.48)	0.54 (0.50-0.58)	0.55 (0.50-0.61)	0.47 (0.43-0.50)
	Risk due to	0.89 (0.85-0.92)	0.74 0.69-0.78)	1.00 (0.95-1.05)	0.94 (0.90-0.99)	0.77 (0.72-0.82)
**Family size**	Small	Ref.	Ref.	Ref.	Ref.	Ref.
	Medium	1.24 (1.21-1.26)	1.26 (1.22-1.31)	1.21 (1.17-1.25)	1.38 (1.34-1.43)	1.13 (1.09-1.16)
	Large	0.85 (0.82-0.88)	0.87 (0.82-0.91)	0.84 (0.80-0.88)	0.90 (0.85-0.95)	0.83 (0.79-0.87)
	No family unit	0.38 (0.36-0.41)	0.37 (0.33-0.40)	0.40 (0.36-0.45)	0.47 (0.42-0.53)	0.34 (0.31-0.37)

ISESI, individual socioeconomic status index; SES, socioeconomic status; Q, quartile.
Overall sample models adjusted for age, sex, type of invitation and health department.
